# The permissive role of TCTP in PM_2.5_/NNK-induced epithelial–mesenchymal transition in lung cells

**DOI:** 10.1186/s12967-020-02256-5

**Published:** 2020-02-11

**Authors:** Li-Zhong Liu, Menghuan Wang, Qihang Xin, Bowen Wang, George G. Chen, Ming-Yue Li

**Affiliations:** 1 Guangdong Provincial Key Laboratory of Regional Immunity and Diseases, Department of Physiology, School of Medicine, Shenzhen University Health Science Center, Shenzhen University, Shenzhen, China; 2 Department of Surgery, The Chinese University of Hong Kong, Prince of Wales Hospital, Shatin, N.T. Hong Kong; 3 Guangzhou Regenerative Medicine and Health Guangdong Laboratory, Guangzhou, China; 4 Shenzhen Research Institute, The Chinese University of Hong Kong, Shenzhen, Guangdong China

**Keywords:** Lung carcinogens PM_2.5_/NNK, Translationally controlled tumor protein (TCTP), Epithelial–mesenchymal transition (EMT), vimentin, microRNA

## Abstract

**Background:**

Translationally controlled tumor protein (TCTP) is linked to lung cancer. However, upon lung cancer carcinogens stimulation, there were no reports on the relationship between TCTP and lung cell carcinogenic epithelial–mesenchymal transition (EMT). This study was designed to investigate the molecular mechanism of regulation of TCTP expression and its role in lung carcinogens-induced EMT.

**Methods:**

To study the role of TCTP in lung carcinogens [particulate matter 2.5 (PM_2.5_) or 4-methylnitrosamino-l-3-pyridyl-butanone (NNK)]-induced EMT, PM_2.5_/NNK-treated lung epithelial and non-small cell lung cancer (NSCLC) cells were tested. Cell derived xenografts, human lung cancer samples and online survival analysis were used to confirm the results. MassArray assay, Real-time PCR and Reporter assays were performed to elucidate the mechanism of regulation of TCTP expression. All statistical analyses were performed using GraphPad Prism version 6.0 or SPSS version 20.0.

**Results:**

Translationally controlled tumor protein and vimentin expression were up-regulated in PM_2.5_/NNK-treated lung cells and orthotopic implantation tumors. TCTP expression was positively correlated with vimentin in human NSCLC samples. Patients with high expression of TCTP displayed reduced overall and disease-free survival. TCTP overexpression could increase vimentin expression and promote cell metastasis. Furthermore, PM_2.5_/NNK stimulation brought a synergistic effect on EMT in TCTP-transfected cells. TCTP knockdown blocked PM_2.5_/NNK carcinogenic effect. Mechanically, PM_2.5_/NNK-induced TCTP expression was regulated by one microRNA, namely miR-125a-3p, but not by methylation on TCTP gene promoter. The level of TCTP was regulated by its specific microRNA during the process of PM_2.5_/NNK stimulation, which in turn enhanced vimentin expression and played a permissive role in carcinogenic EMT.

**Conclusions:**

Our results provided new insights into the mechanisms of TCTP regulatory expression in lung carcinogens-induced EMT. TCTP and miR-125a-3p might act as potential prognostic biomarkers and therapeutic targets for NSCLC.

## Background

Smokers under exposure to cigarette and non-smokers without history of tobacco smoking are estimated to account for approximately 75% and 25% of all lung cancers respectively [1]. Among the numerous carcinogenic agents in tobacco products, 4-methylnitrosamino-l-3-pyridyl-butanone (NNK) was a major contributor to non-small cell lung cancer (NSCLC) cell carcinogenesis and the molecular mechanism involved has been well studied [2]. Among the factors that contributed to the development of lung cancer in never smokers (LCNS), polluted air, especially particulate matter 2.5 (PM_2.5_), played the main role in lung carcinogenesis [3–5]. We recently demonstrated that PM_2.5_ could work similarly to NNK in regulating lung cell proliferation, migration, invasion, and cancer stem cell formation by inhibiting 15-LOX1/15-LOX2 [6–8].

Cells undergoing epithelial–mesenchymal transition (EMT) acquired cellular movement by losing cell polarity, repressing expression of various cytoskeletal proteins such as E-cadherin while promoting expression of mesenchymal proteins such as vimentin and N-cadherin [9]. Cancer stem cell (CSC) plasticity and cancer dissemination in the metastatic process were associated with EMT [10]. The phenotypic changes that characterized the transition from CSCs to differentiated cancer cells involved a process occurring in EMT [11]. It has been demonstrated that EMT was the link between benign lung diseases and lung carcinogenesis. Thus, EMT played a central role in the development of lung cancer [11].

Translationally controlled tumor protein (TCTP) was a highly conserved protein initially discovered in mouse tumor cells [12]. TCTP was a multifunctional protein implicated in a diversity of biological processes including cell and tumor proliferation [13–15]. It was over-expressed in various malignancies including lung cancer [16, 17]. Depletion of TCTP in colon cancer cell significantly reduced cell metastasis [18]. Recent findings established TCTP as an EMT inducer by GSK3β pathway in porcine renal proximal tubule cell line [19]. TCTP was a target of TGF-β1 as a key regulator of EMT in A549 cell line [17]. The above findings indicated that TCTP might be involved in carcinogenesis of different tissues, including the lung. However, upon lung cancer carcinogens stimulation, there was no report on the relationship between TCTP expression and cellular EMT. Furthermore, the molecular mechanism of TCTP regulation was unknown in this process. The present study aimed to investigate the role of TCTP in NNK/PM_2.5_-induced EMT and how TCTP expression was regulated during this process. Cell derived xenografts and human lung cancer samples were utilized for confirmation.

## Methods

### Reagents

Fetal bovine serum (FBS), Cell Dissociation Reagent, medium LHC-9 and Dulbecco’s modified Eagle medium (DMEM) were provided by Invitrogen (Carlsbad, CA). The antibody against VECTOR ImmPRESS Anti-Goat Ig was provided by Santa Cruz Biotechnology (Santa Cruz, CA). Protein Block solution and DAB substrate were provided by Abcam (Cambridge, MA). Antibodies against vimentin, E-cadherin, N-cadherin and TCTP were purchased from Cell Signaling (Boston, MA). ECL reagent kit was purchased from GE Healthcare Life Sciences (Piscataway, NJ). NNK was purchased from Chemsyn Science Laboratories (Lenexa, KS). 10 µM NNK was used in the experiments [20]. PM_2.5_ was collected at Kowloon Tong of Hong Kong and 5 µg/ml PM_2.5_ was used in the experiments as details described in our previous study [21].

### PM_2.5_ sample extraction

The PM_2.5_ water extraction was prepared [22]. In brief, the filters were cut into pieces and soaked in 8 ml of Milli-Q water in 15 ml metal free centrifuge tubes, then extracted by vortex-assisted shaking using a multi-tube vortex mixer (Model X-2500, VWR). After 12 h of vortex, the extracts were filtered with 0.22 μm filter membranes and stored at − 20 °C.

### Cell culture

Lung cancer cell line NCI-H23 was purchased from the American Type Culture Collection (ATCC). Bet1A is lung normal bronchial epithelial cells infected with SV40 virus (gift of J. E. Lechner, Laboratory of Human Carcinogenesis, National Cancer Institute). Bet1A cells are useful for studies of multistage bronchial epithelial carcinogenesis because the cells retained features of epithelial cells with the presence of epithelial marker cytokeratin and no tumors formed after s.c. injection of the cells in nude mice [23–26]. NCI-H23 cells were cultured in DMEM supplemented with 10% inactivated FBS. Bet1A cells were cultured in medium LHC-9. In order to better display and maintain a EMT state, NCI-H23 and Bet1A cells were used for different experiments after the cells were cultured in the presence of 5 µg/ml PM_2.5_ or 10 µM NNK for 15 or 28 days. Non-treatment cells were set up as the control.

### Human lung tissues

In total, 109-paired human primary non-small cell lung cancers (NSCLCs) and adjacent normal lung tissues were collected immediately after surgical resection at the Prince of Wales Hospital (Hong Kong, China). The study was performed in accordance with the ethical principles and guidelines for human research of the Helsinki Declaration, and human ethics approval (2014.649 and 2015.729) was obtained from the joint Chinese University of Hong Kong-New Territories East Cluster Clinical Research Governance and Management Committee. An informed consent for human tissues for research purposes only was obtained from all patients recruited. Tumor tissue samples were taken from the central part of the tumors. Of the 109 patients, 34 were current cigarette smokers with an average smoking history of 35 years, 36 patients were previous cigarette smokers with an average smoking history of 28 years, and the other 39 patients were non-smokers. All tumor and non-tumor tissue specimens were confirmed by histological examination. The specimens were snap-frozen in liquid nitrogen and stored at − 80 °C and were also fixed in 10% formalin and embedded in histochemical staining examination.

### The OS (overall survival) and RFS (recurrence-free survival) analysis by Kaplan–Meier plotter software

Based on online survival analysis software: Kaplan–Meier plotter (http://kmplot.com/analysis/), the OS of the two groups of patients (n = 1926) with high and low TCTP expression in NSCLC tissues and the RFS of the two groups of patients (n = 726) with high and low TCTP expression in NSCLC tissues were calculated respectively.

### Western-blot

The cultured cells were collected after the respective treatment. The protein was isolated and western blot was performed as described previously [20].

### Immunohistochemistry

Immunohistochemical staining of TCTP and vimentin were performed for 109 pairs of human lung tissues as described previously [7].

### Xenograft model

Subcutaneous (S.C.) tumor model was set up in nude mice to determine the TCTP and EMT related proteins expression in tumorigenic lung cells treated with PM_2.5_ or NNK for 28 days. All animal experiments were conducted in accordance with the Animals (Control of Experiments) Ordinance Chapter 340, and approved (15-210-GRF) by the Animal Experimentation Ethics Committee of CUHK. The establishment of the mouse tumor model was performed as our previous publication [27]. Female BALB/C athymic nude mice were housed in groups of five and given 5 days to acclimate to the housing facility—SPF, a temperature-regulated environment (20 °C) and humidity of 50% with a reversed 12:12 h light–dark cycle. Briefly, NCI-H23 cells (5 × 10^6^) were S.C. implanted into the left and right dorsal flank of 5-week-old female BALB/C athymic (nu/nu, n = 9/group, randomised group) nude mice, using a 1 ml syringe with a 25G needle attached, respectively. After cell implantation, the general health (including vital sign, food intake, body weight, and activity of the mice) were monitored daily. No adverse events were observed. Tumors were measured in two dimensions by external caliper and Tumor volume (V) was estimated by measuring the longest diameter (L) and shortest diameter (W) of the tumor and calculated by formula [length × width (mm)^2^]/2 [27]. The size of tumor was monitored for 6 weeks. At the endpoint, the mice were sacrificed by cervical dislocation while under using overdose of sodium pentobarbital and tumors were harvested and measured.

### MassArray for methylation assay

MassArray for methylation assay (BGI, China) of genes was employed to detect the TCTP gene promotor methylation levels. The software, http://www.ebi.ac.uk/Tools/seqstats/emboss_cpgplot/, was used to predict the CpG islands from the upstream of 5000 bp of start codon to downstream of 1000 bp start codon of genes. Sequenom^®^EpiDesigner process was used to design plans for gene methylation assay.

### Real-time PCR

Total RNA including miRNA was purified with the miRNeasy Mini Kit (#217004, Qiagen MD). The expression of miR-27a, miR-27b, miR-33a, miR-33b, miR-128, miR-125-3p, miR-371a-5p, miR-365, miR-425 (# E01007) and U6 (#E01008) was detected using Hairpin-it™ miRNAs qPCR Quantitation Kit (Genepharma, China). miR-125a-3p mimic (YM00471085-ADA, #339173 miRCURY LNA miRNA Mimic, Qiagen MD), miR-425 mimic (YM00471725-ADA, #339173 miRCURY LNA miRNA Mimic, Qiagen MD) miR-371-5p mimic (YM00472003-ADA, #339173 miRCURY LNA miRNA Mimic, Qiagen MD) and unrelated sequences miR-NC used as negative controls (YM00479902-ADA, #339173 miRCURY LNA miRNA Mimic, Qiagen MD) were provided by Qiagen. The expression of miR-125-3p, miR-371a-5p, and miR-425 in the mimic microRNAs transfected cells were detected by miRCURY LNA miRNA PCR Starter Kit (#339320, Qiagen MD). The expression of target miRNAs in the human lung tissues, nude mice tumors, or the treated and control cells was normalized using U6 or miR-NC controls and the fold change in the expression of each target gene was calculated.

### Reporter assays of 3′-UTR-luciferase plasmid of TCTP

The Luc-TCTP-WT with full-length 3′-untranslated region (UTR) of TCTP (217 nt) were cloned into pGL3-promoter-vector (Additional file 1: Figure S3A–C). The following primer sequences were used: TCTP-UTR_EcoRI-Forward GGTG′AATTCCAAATGTGGCAATTATTTTGG and TCTP-UTR_AgeI-Reverse CCTA′CCGGTCTCTCAAATGAGTTTAAATGC. NCI-H23 and Bet1A cells were treated with PM_2.5_ or NNK for 28 days. Non-treated cells were set up as control. Reporter assays were performed using the Dual-luciferase assay system (Promega, Madison, WI), normalized for transfection efficiency by co-transfected Renilla luciferase.

### Wound healing assay

To assess cell motility, NCI-H23 cells or Bet1A cells under different treatments (5 × 10^5^ cells/ml) were seeded in 24-well plates (Corning, New York). Non-treated cells were set up as the control. The cells were transfected with plasmids for 24 h. The monolayers of NCI-H23 were scraped with a sterile 1000-µl micropipette tip (0 h) and Bet1A were scraped with a sterile 200-µl micropipette tip (0 h) to create a denuded zone with a constant width and were washed twice with phosphate-buffered saline (PBS) to remove cellular debris. The cells were cultured in culture medium without FBS. The scratched monolayers were imaged for 24 h or 48 h using an inverted microscope (Olympus, Japan) at 200× magnification in a blinded fashion. The relative percentage of wound healed was analysis by Image J software.

### Invasion assay

Cell invasion was determined using BD BioCoat Matrigel Invasion Chamber (BD Biosciences). Briefly, NCI-H23 cells or Bet1A cells under different treatments (2 × 10^4^ cells/well) were seeded onto the top chamber in serum-free cell culture medium. Complete culture medium (supplemented with 10% FBS) was added to the bottom chamber as a chemoattractant. After 48 h, cells that had invaded through the membrane were stained with 0.1% Crystal violet. Migrated cells in randomly selected fields were observed by light microscopy (Olympus, Japan).

### Plasmid DNA and transfection

The complementary DNAs for wild-type human TCTP plasmid pEGFP-TCTP, pSicoR-TCTP shRNA and control vectors were gifted from Prof. Ying Ming (College of Life Sciences and Oceanography, Shenzhen University). The FuGENE HD transfection reagent (Roche, Basel, Switzerland) was used to transfect plasmids into cells according to the manufacturer’s instructions. Cells transfected with the empty vectors were used as the control.

### Statistical analysis

Continuous data was expressed as mean ± SD (continuous variables) or described as frequency and percentage (categorical data). The difference was determined by ANOVA with repeated-measures ANOVA. To compare the difference between two groups, independent sample t test, paired t-test, or Mann–Whitney U test was used. Based on the TCTP/vimentin expression levels in tumor tissues and the paired non-tumor tissues, the expression level was graded. When the expression of TCTP and vimentin level in each paired sample was considered, the expression in non-tumor tissue was set up as the normal, and the expression in tumor tissue was graded as low/high expression in comparison with the non-tumor tissue. The clinic-pathologic features in patients with relative expressing TCTP/vimentin were compared using Pearson’s Chi-squared test or Fisher’s exact test for categorical variables. All the statistical analyses were performed using GraphPad Prism, version 6.0 (GraphPad Software) or SPSS, version 20.0 (SPSS Inc.). Kaplan–Meier plots and log-rank test were used for survival analysis. p < 0.05 was considered statistically significant.

## Results

### TCTP expression was related to PM_2.5_ and NNK induced EMT

**Fig. 1:**
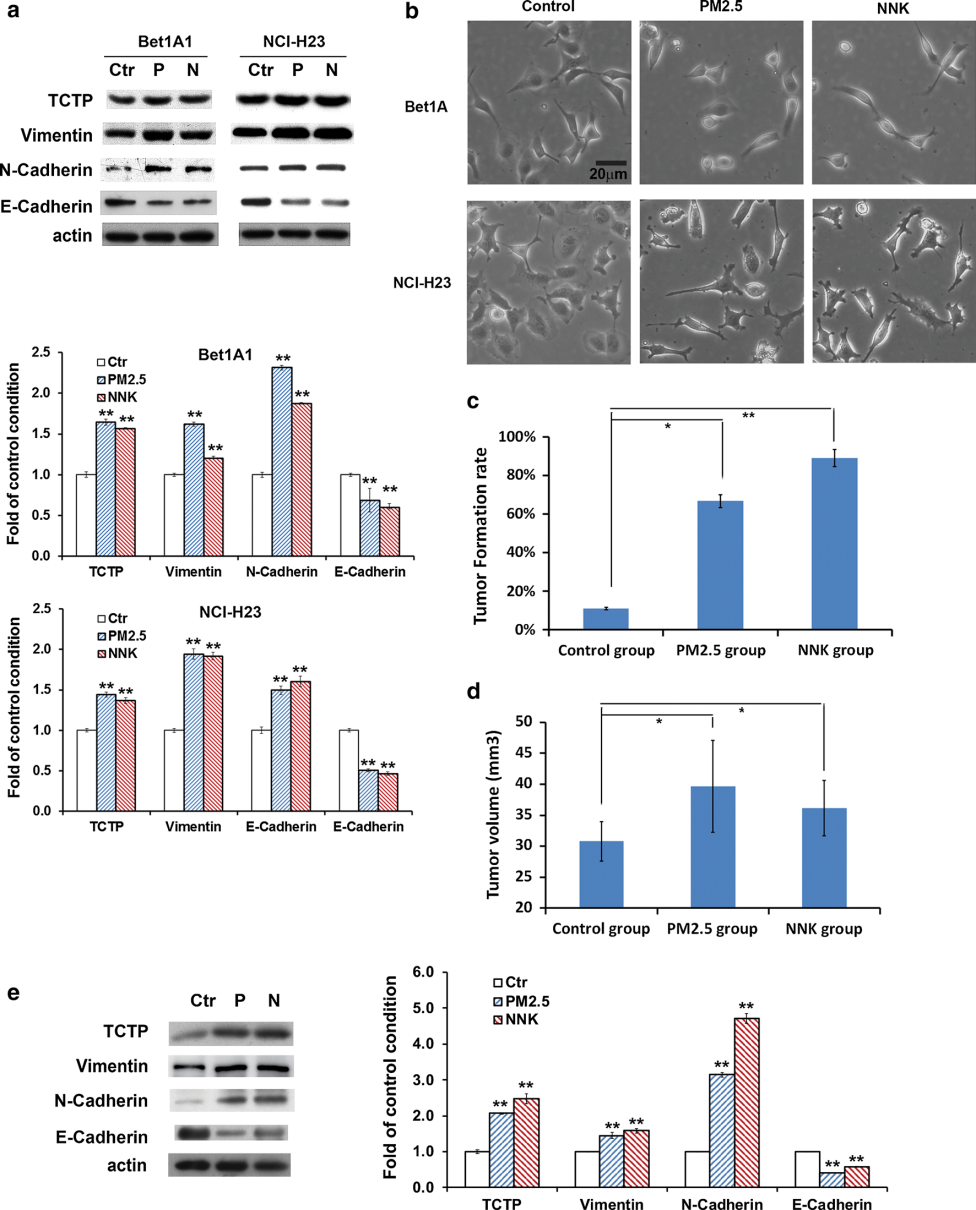
Both TCTP expression and EMT were involved in PM_2.5_- and NNK-induced lung carcinogenesis. **a** PM_2.5_ or NNK treatment promoted TCTP expression and EMT. Cells were treated with 5 µg/ml PM_2.5_ or 10 µM NNK for 28 days. Cells without treatment were the control condition. Proteins expression was examined as indicated. The quantification of protein was carried out by densitometry analysis, and the result was presented by the relative intensity of the control condition based on actin normalization for total protein. The relative intensity of protein bands was summarized by column figure. The values indicate the mean ± SD of three independent experiments (Ctr: non-treatment control; P: PM2.5; N: NNK; *p < 0.05 and **p < 0.01 vs Ctr. **b** PM_2.5_ and NNK treatment induced mesenchymal transformation of the cells. Cells were treated by PM_2.5_ or NNK for 28 days. Non-treated cells were set up as control. Images were taken using phase contrast microscope (Nikon) (scale bar, 20 μm). **c** Tumor formation assay and **d** tumorigenicity assay in nude mice. NCI-H23 cells untreated or treated by PM_2.5_ and NNK respectively for 28 days were S.C. implanted into the nude mice. Tumor formation rate was calculated after 2 weeks. Data are the average rate expressed as the mean ± SD of nine mice. The growth of tumors as indicated by tumor volume was detected after 6 weeks. Data are the average tumor volume expressed as the mean ± SD of eight mice. *p < 0.05 and **p < 0.01 vs control group. **e** Detection of TCTP and EMT markers in the xenografts of NCI-H23 cells. After 6 weeks of injection, three of each group of tumor tissue proteins from the xenografts were pooled respectively and examined as indicated. The quantification of protein was carried out by densitometry analysis, and the result was presented by the relative intensity of the control condition based on actin normalization for total protein expressed. The relative intensity of protein bands was summarized by column figure. The values indicate the mean ± SD of three independent experiments. *p < 0.05 and **p < 0.01 vs control group

As shown in Fig. 1a, b, TCTP expression was up-regulated accompanied with cells exhibiting scattered, elongated, and mesenchymal-like morphology under PM_2.5_/NNK treatment. EMT acquired cellular movement by the loss of cell polarity, repression of various cytoskeletal proteins such as E-cadherin and expression of mesenchymal proteins such as vimentin and N-cadherin [9]. Consistently, the expression of E-cadherin was greatly reduced, whereas vimentin and N-cadherin were significantly elevated by PM_2.5_/NNK treatment (Fig. 1a). The results revealed that TCTP was positively correlated with lung carcinogens-induced carcinogenesis.

We evaluated the expression of TCTP and EMT markers in cell derived xenografts. NCI-H23 cells pre-treated with PM_2.5_ or NNK or not were injected into nude mice. The tumor formation rate/tumor growth in the treated groups was higher/faster than the non-treated control group (Fig. 1c, d). Compared with the control group, there was significant increase in the expression of TCTP, vimentin and N-cadherin, while the expression of E-cadherin reduced (Fig. 1e). Both in vivo and in vitro results suggested the elevated TCTP expression was involved in PM_2.5_/NNK-induced EMT during lung carcinogenesis. Here, the epithelial Bet1A cells could not form tumors in nude mice probably because they still retained some features of epithelial cells even after PM_2.5_ and NNK stimulation.

### TCTP and vimentin expression was up-regulated and positive correlation with each other in human NSCLC tumors

**Fig. 2:**
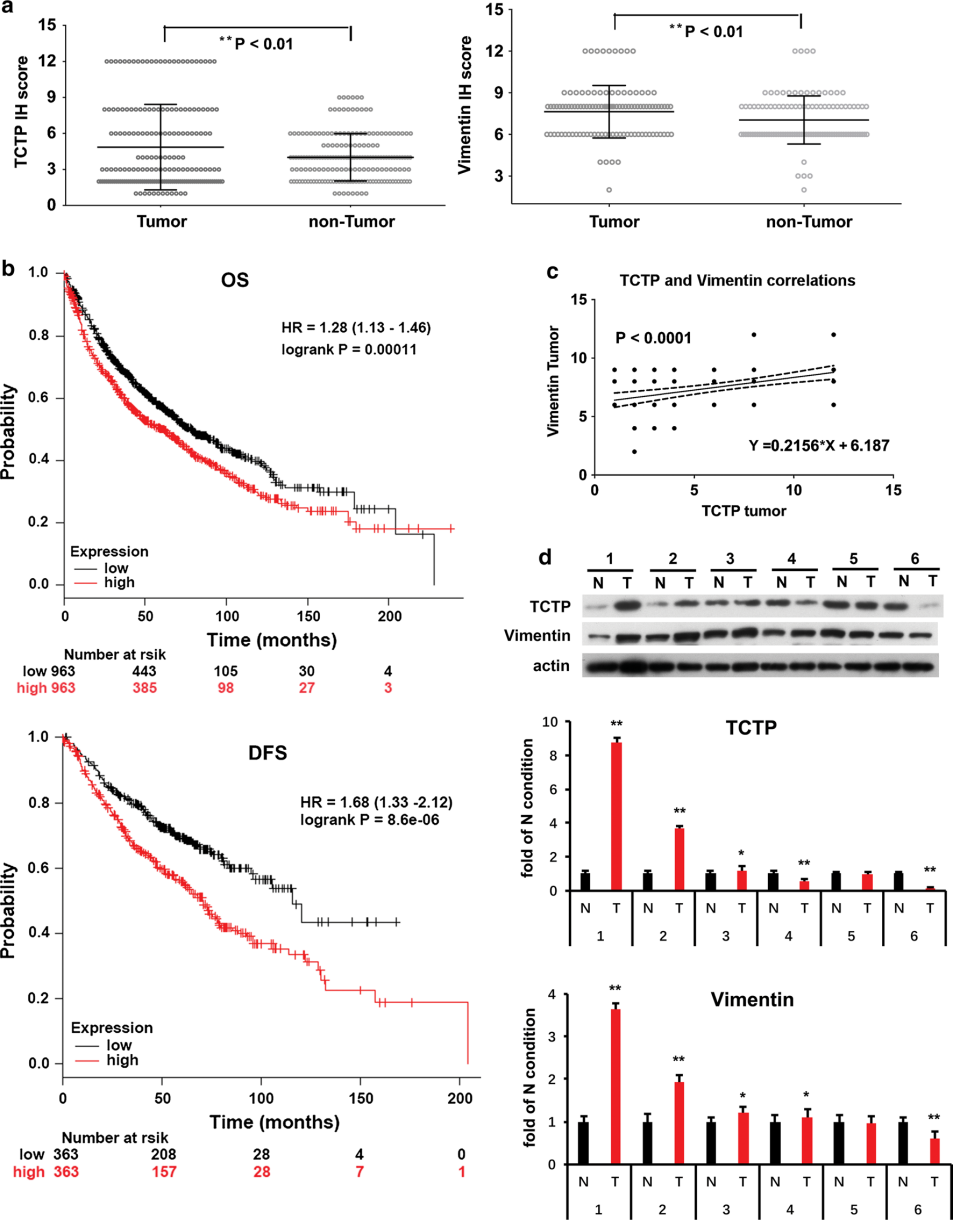
The expression and correlation of TCTP and vimentin in human lung tumor tissues. **a** The levels of TCTP and vimentin in 109 paired NSCLC tissues and adjacent normal tissues. The stained tissues were examined using the Zeiss Spot imaging system and the immunohistochemical staining scored was assessed and expressed as Mean with range. Wilcoxon signed ranks test was used to compare the values between tumor tissues and non-tumor tissues. **b** Negative relationship of TCTP with lung cancer patient outcomes on OS (overall survival) and RFS (recurrence-free survival). The OS of the two groups of patients (n = 1926) with high and low TCTP expression in NSCLC and the RFS of the two groups of patients (n = 726) with high and low TCTP expression in NSCLC tissues were analyzed based on online survival analysis software: Kaplan–Meier plotter. TCTP high level is significantly related to poor OS and poor RFS in lung cancer patients. **c** Positive correlation of TCTP and vimentin expression in lung tumor. The correlation of expression levels of TCTP and vimentin in 109 NSCLC tumor tissues scored were analyzed. A positive correlation between TCTP and vimentin expression level in lung tumor were shown. **d** TCTP and vimentin expression in human lung tumor tissues and non-tumor tissues. TCTP and vimentin protein levels were examined in 6 randomly selected paired NSCLC specimens. The quantification of protein was carried out by densitometry analysis, and the result was presented by the relative intensity of each of non-tumor condition based on actin normalization for total protein. The relative intensity of protein bands was summarized by column figure. The values indicate the mean ± SD of three independent experiments (N stands for non-tumor tissue, T stands for tumor tissue; *p < 0.05 and **p < 0.01 vs. N condition). 3 out of 6 and 4 out of 6 of patients showed higher levels of TCTP and vimentin respectively in tumor tissues than that in adjacent normal tissues. A positive correlation between TCTP and vimentin level in 5 patients’ lung tumor were found

Immunohistochemistry (IHC) and immunoreactivity scoring results showed that TCTP and vimentin were highly expressed respectively in 68/109 (62.4%) and 61/109 (56.0%) of NSCLCs and the overall expressions of both were up-regulated significantly in cancer tissues than in normal adjacent tissues (Fig. 2a and Additional file 1: Table S1). Correlation analysis showed there was no significant association of TCTP levels with age, gender, smoking status, histology, and pathology state. However, the high level of TCTP was significantly associated with tumor size (Additional file 1: Table S1). Patients with higher expression of TCTP displayed reduced overall and disease-free survival (Fig. 2b). Collectively, TCTP was negatively associated with the clinical outcomes including survival rates and recurrence of lung cancer. The high level of vimentin was significantly associated with age but not with other clinicopathological features (Additional file 1: Table S1). TCTP and vimentin expression showed no significant difference in smoker, ex-smoker and non-smoker patients (Additional file 1: Table S1), implicating that they might act as tumor enhancers for NSCLCs in both smokers and non-smokers. Vimentin was the well-characterized biomarker of EMT during lung carcinogenesis [9]. The high expressions of TCTP and vimentin were positive-related in NSCLC tumors (Fig. 2c). In 6 randomly selected paired NSCLC specimens, the expression of TCTP and vimentin was both up-regulated in 3 patients’ lung tumor and a positive correlation between TCTP and vimentin level in 5 patients’ lung tumor was found (Fig. 2D).

### TCTP controlled vimentin expression and mediated PM_2.5_- and NNK-induced metastasis of lung cells

**Fig. 3:**
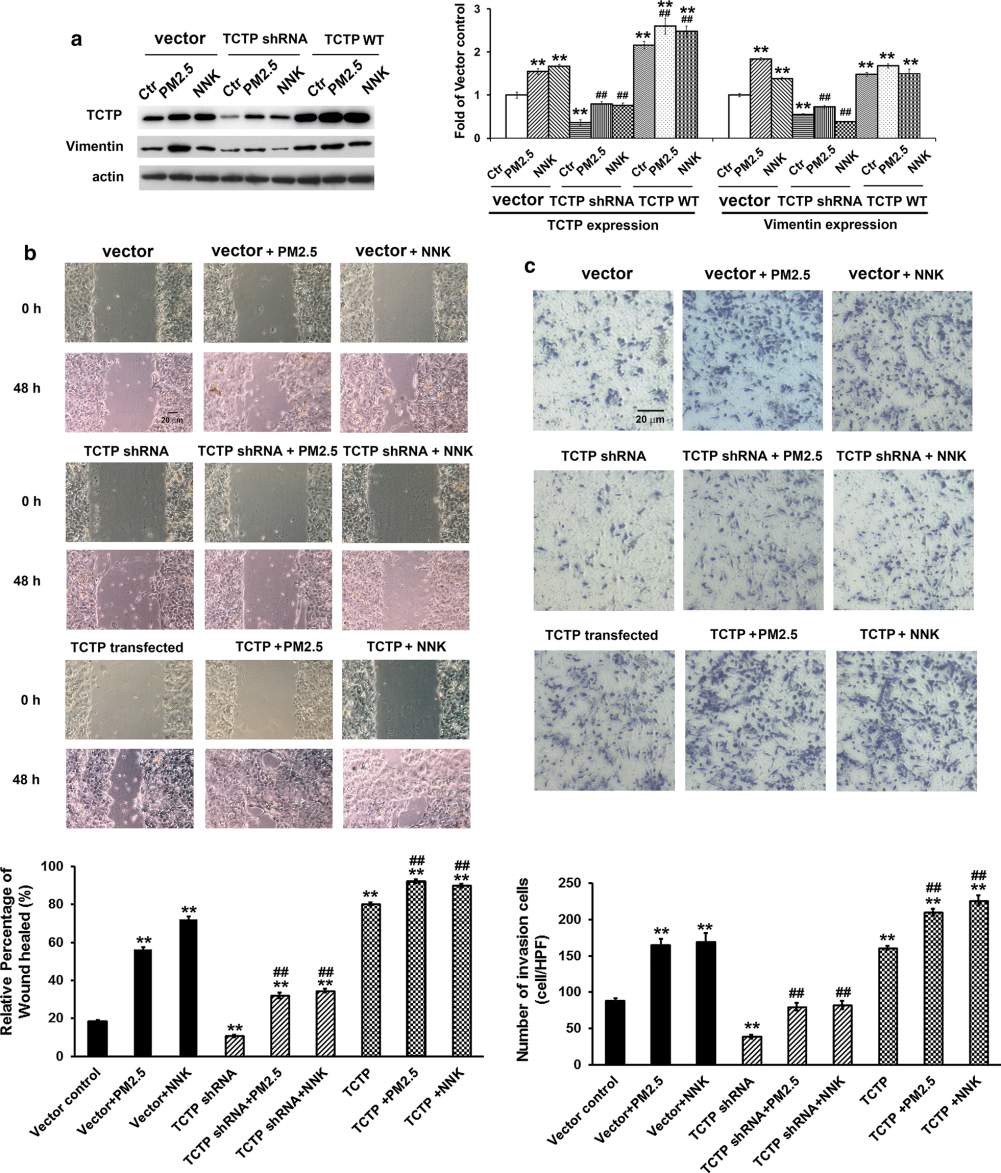
TCTP controlled vimentin expression and mediated PM_2.5_- and NNK-induced metastasis of lung cells. **a** TCTP controlled vimentin expression. Bet1A cells were first treated with PM_2.5_ or NNK for 28 days, then the cells were co-transfected with scramble shRNA pSicoR vector + empty pEGFP vector or cells were transfected with pSicoR vector containing TCTP shRNA or pEGFP vector containing TCTP gene respectively. Cells were incubated for another 24 h. The levels of TCTP and vimentin were determined as indicated. The equal loading was confirmed by measuring actin protein. The quantification of protein was carried out by densitometry analysis, and the result was presented by the relative intensity of the control condition based on actin normalization for total protein expressed. The relative intensity of protein bands was summarized by column figure. The values indicate the mean ± SD of three independent experiments (**p < 0.01 vs vector control; ^##^*p *< 0.01 vs vector + PM_2.5_ or vector + NNK respectively). **b** TCTP was required for PM_2.5_- or NNK-induced cell migration. Bet1A cells were treated with PM_2.5_ or NNK for 28 days. Then the cells were transfected with empty vector or vector contained TCTP gene or TCTP shRNA respectively, and cells were incubated for another 48 h. Cell migration was detected by wound-healing assay. Images were taken using phase contrast microscope (Nikon) (scale bar, 20 μm). The relative percentage of wound healed was expressed as mean ± SD of three independent experiments. **p < 0.01 vs vector control; ^##^*p *< 0.01 vs vector + PM_2.5_ or vector + NNK respectively. **c** TCTP was required for PM_2.5_- or NNK-induced cell invasion. Bet1A cells were treated by PM_2.5_ or NNK for 28 days. Then the cells transfected with vectors as indicated and incubated for another 48 h. Cell invasion was detected by trans-well experiment. Images were taken using phase contrast microscope (Nikon) (scale bar, 20 μm). The numbers of invading cells in four randomly selected high-power fields (HPF) were counted and the average number of cells in a HPF was calculated. The values indicate the mean ± SD of three independent experiments. **p < 0.01 vs vector control; ^##^*p *< 0.01 vs vector + PM_2.5_ or vector + NNK respectively

We further checked the roles of TCTP in EMT and its relationship with vimentin. Effects of TCTP overexpression or knockdown were examined in Bet1A cells (Fig. 3) and NCI-H23 cells (Additional file 1: Figure S1B–D) treated with PM_2.5_ or NNK respectively. TCTP shRNA/scramble shRNA were cloned into pSicoR vector and TCTP gene was cloned into pEGFP vector. Thus, cells co-transfected with scramble shRNA (in pSicoR vector) and empty pEGFP were set as vector control. Compared with non-transfected cells, the expressions of TCTP and vimentin did not change after vectors transfection (Additional file 1: Figure S1A). The expression of both TCTP and vimentin was enhanced when lung cells were treated with PM_2.5_ or NNK (Fig. 3a and Additional file 1: Figure S1B). TCTP overexpression promoted vimentin expression, which mimicked the effect of PM_2.5_/NNK treatment. When TCTP was knocked down, vimentin expression was inhibited significantly (Fig. 3a and Additional file 1: Figure S1B). Compared with control group, TCTP overexpression promoted cell migration and invasion significantly whereas TCTP knockdown showed counteraction effect (Fig. 3b, c and Additional file 1: Figure S1C, D). The results demonstrated that TCTP was indispensable during PM_2.5_ and NNK induced lung carcinogenic EMT. Indeed, when TCTP was knocked down by its specific shRNA in NCI-H23 cells followed by the CDX experiment, no tumors were found in the nude mice.

### TCTP expression was regulated by MicroRNAs rather than DNA methylation in the process of PM_2.5_/NNK stimulation

**Fig. 4:**
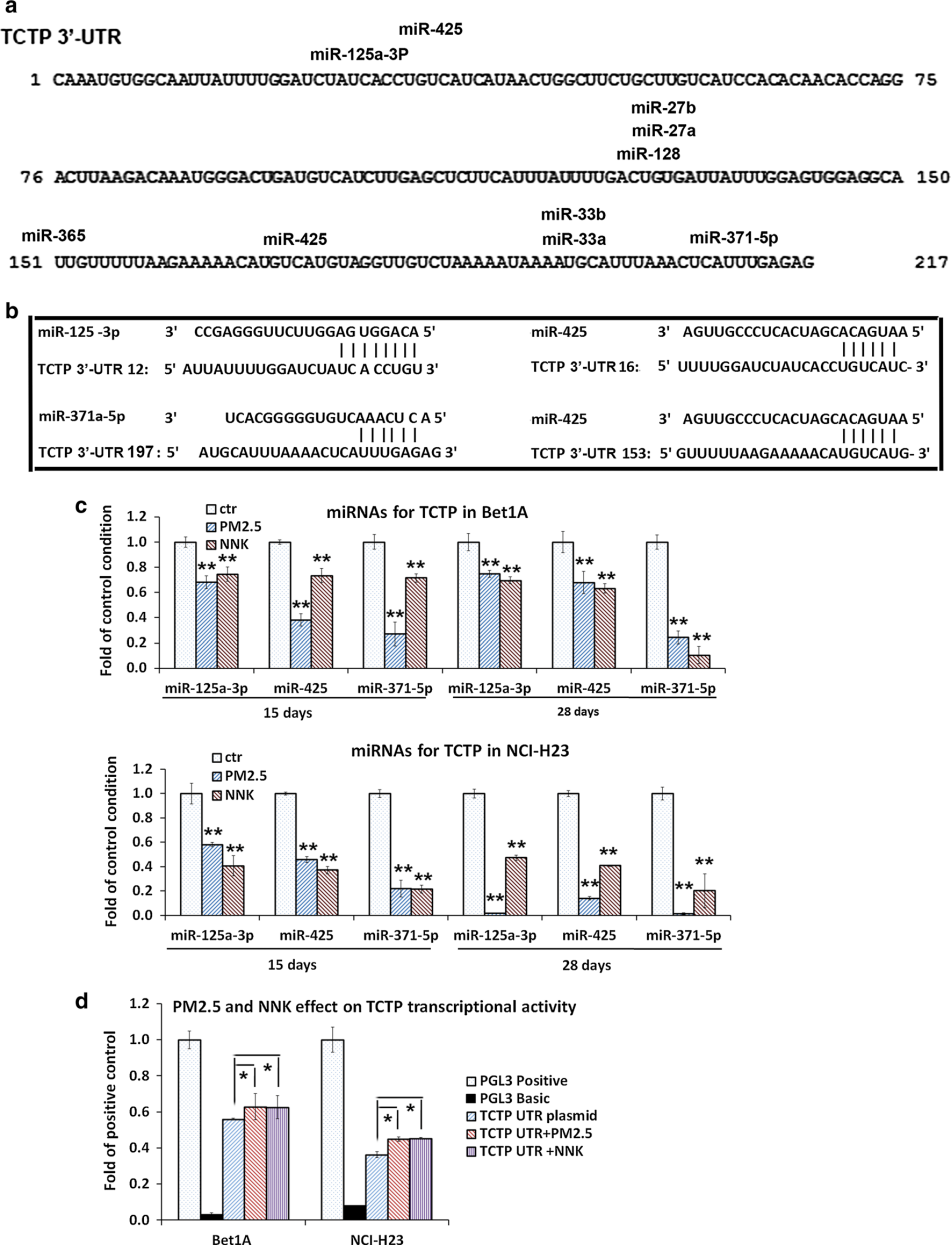
MicroRNAs targeted on TCTP were down-regulated by PM_2.5_ and NNK. **a** Predicted binding sites of miRNAs on TCTP 3′-UTR region. All the individual candidate miRNAs were reported, namely miR-27a, miR-27b, miR-33a, miR-33b, miR-128, miR-125a-3p, miR-371a-5p, miR-365 and miR-425. The predicted binding sites of miRNAs on TCTP 3′-UTR were shown. **b** Predicted binding sites of miR-125a-3p, miR-371a-5p, and miR-425 on TCTP 3′-UTR region. The alignment of miR-125a-3p, miR-371a-5p, and miR-425 with four predicted binding sites of TCTP 3′-UTR were shown. **c** MiRNAs targeted on TCTP 3′-UTR region were down-regulated by PM_2.5_ and NNK. Bet1A and NCI-H23 cells were treated with PM_2.5_ or NNK for 15 days and 28 days respectively. Total RNAs were extracted for real-time PCR assay. The values indicate the mean ± SD of three independent experiments (**p < 0.01 vs control). **d** TCTP transcriptional activity was upregulated by PM_2.5_ or NNK. Bet1A cells and NCI-H23 cells were treated with PM_2.5_ or NNK for 28 days. Then cells were transfected with luciferase reporter constructs containing the pGL3-TCTP 3′-UTR and incubated for 24 h. The pGL3 basic vector and the pGL3 control were used as negative and positive controls respectively. Reporter assays were performed using the Dual-luciferase assay system, normalized for transfection efficiency by co-transfected Renilla luciferase. Data are expressed as mean ± SD of three independent experiments performed in triplicate (*p < 0.05 vs TCTP 3′-UTR)

It has been reported that TCTP expression was regulated at both transcription and translation levels in cells [28], we hypothesized that the expression of TCTP could be regulated by microRNAs or/and by promotor methylation when lung cells were exposed to PM_2.5_ and NNK. Predictions of microRNA candidates were made based on the 3′ UTRs of the TCTP gene using two bioinformatics tools: the mirSVR predicted target site scoring method [29] and the online tools at MicroRNA.org (http://www.microrna.org). All the individual candidate miRNAs were listed (Fig. 4a). Expressions of these nine miRNAs were detected in Bet1A and NCI-H23 cells treated with PM_2.5_ or NNK for 15 days and 28 days respectively. Three miRNAs, miR-125a-3p, miR-425 and miR-371-5p, which potentially targeted on TCTP 3′-UTR region (Fig. 4b), were significantly decreased in both Bet1A and NCI-H23 cells after PM_2.5_ or NNK treatment (Fig. 4c). In consistence with these results, the transcriptional activities of TCTP 3′-UTR reporter was significantly upregulated in PM_2.5_-/NNK-treated lung cells compared with those in control cells (Fig. 4d), suggesting that these three miRNAs could function as negative regulators of TCTP during the process of EMT.

For methylation assay, the results showed that the number of methylation points on TCTP promoter were less and the total methylation level was quite low in both cells. Furthermore, the alteration of methylation level of two points (CpG 7 and CpG 40) in NCI-H23 and Bet1A cell showed discrepancy (Additional file 1: Figure S2 and Table S2). It was unlikely that the expression of TCTP was manipulated by methylation in the presence of PM2.5/NNK.

### miR-125a-3p negatively regulated TCTP transcriptional activity

**Fig. 5:**
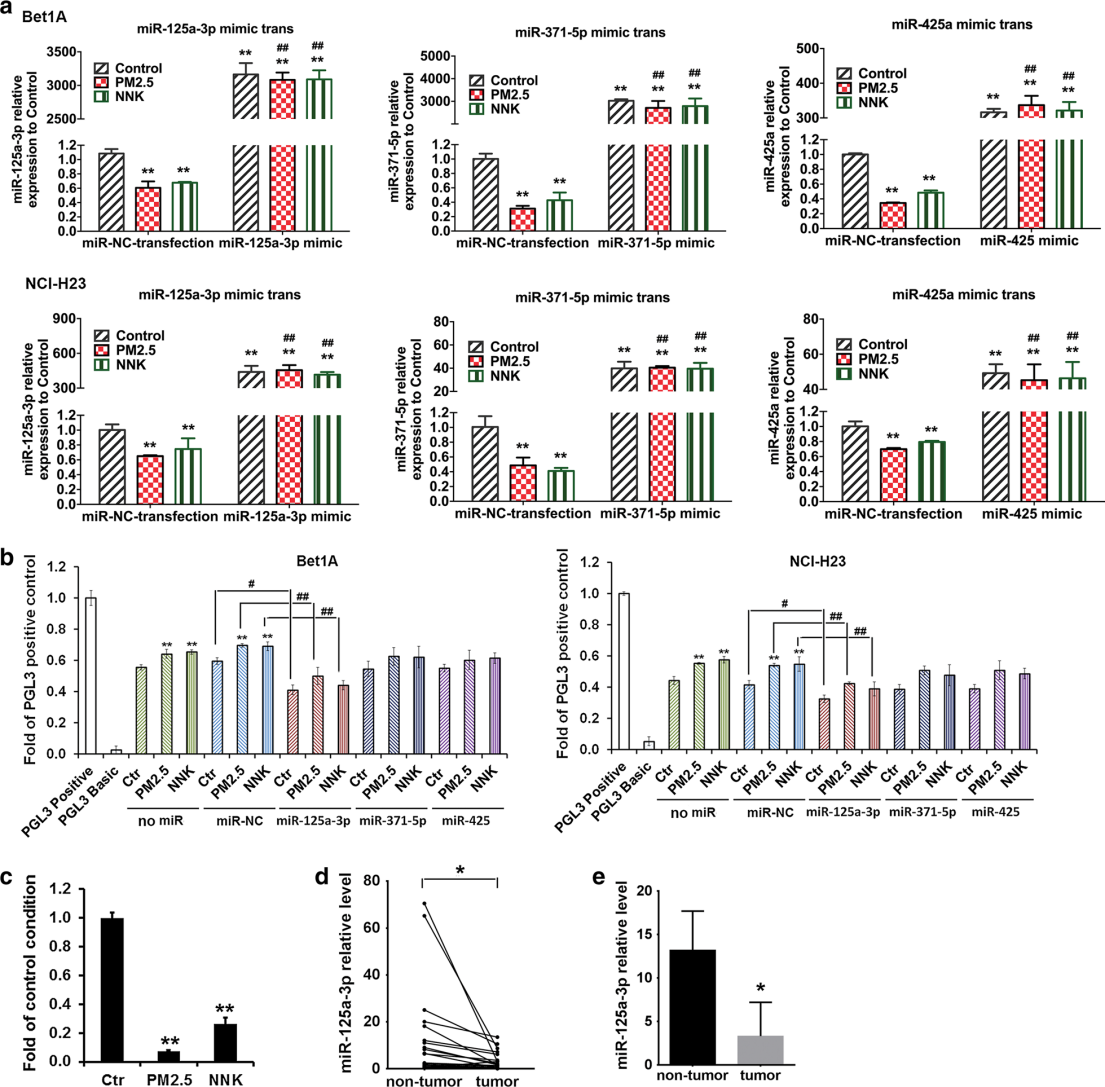
TCTP transcriptional activity was negatively regulated by miRNA. **a** Overexpression of miR-125a-3p, miR-371a-5p, and miR-425 in PM_2.5_- or NNK-induced lung cells. Bet1A cells or NCI-H23 cells were treated with PM_2.5_ or NNK for 28 days. Then the cells were transfected with miR-125a-3p mimic, miR-371a-5p mimic, miR-425 mimic, and miR-NC control respectively for 24 h. Total RNA was extracted for real-time PCR assay. The values indicate the mean ± SD of three independent experiments. (*p < 0.05 and **p < 0.01 vs miR-NC control. ^#^*p *< 0.05 and ^##^*p *< 0.01 vs miR-NC + PM_2.5_ or miR-NC + NNK respectively. **b** TCTP transcriptional activity was downregulated by miR-125a-3p. Bet1A cells and NCI-H23 cells were treated with PM_2.5_ or NNK for 28 days and were con-transfected with miR-125a-3p mimic, miR-371a-5p mimic, miR-425 mimic, and miR-NC respectively with luciferase reporter constructs containing the pGL3-TCTP 3′-UTR. The pGL3 basic vector and the pGL3 control were used as negative and positive controls respectively. Reporter assays were performed using the Dual-luciferase assay system, normalized for transfection efficiency by co-transfected Renilla luciferase. Data are expressed as mean ± SD of three independent experiments performed in triplicate. **p < 0.01 vs no miR control. ^*#*^*p *< 0.05 and ^*##*^*p *< 0.01, when compared between indicated groups. **c** miR-125a-3p expression in the xenografts of NCI-H23 cells. In the CDX experiment, three of each group of tumor tissue RNA from the mice was pooled together and miR-125a-3p expression in the xenografts of NCI-H23 cells was detected by Real-time PCR. The values indicate the mean ± SD of three independent experiments, **p < 0.01 vs control group. **d**, **e** The levels of miR-125a-3p in 20 paired NSCLC tissues and adjacent normal tissues. MiR-125a-3p expression in the human lung tumor tissues and paired non-tumor tissues was examined by Real-time PCR. ( was paired t-test result to compare the miR-125a-3p expression between tumor tissues and non-tumor tissues (*p < 0.05), and **e** was unpaired t-test result to compare the mean of miR-125a-3p expression between tumor tissues and non-tumor tissues as data were expressed as mean ± SE (*p < 0.05)

To investigate whether miR-125a-3p, miR-425 and miR-371-5p could inhibit TCTP expression during PM_2.5_ and NNK induced lung carcinogenesis, we overexpressed miR-125a-3p, miR-425 and miR-371-5p in cells by transfecting the mimics of miR-125a-3p, miR-425 or miR-371-5p or negative control (miR-NC) into the cells. Our data demonstrated that in miR-NC transfected cells, miR-125a-3p, miR-425 and miR-371-5p expression dropped significantly upon PM_2.5_ or NNK treatment (Fig. 5a). When the mimics of miR-125a-3p, miR-425 or miR-371-5p were transfected into the cells respectively, all three miRNAs were highly expressed, which compensated the down-regulated effect of the carcinogens (Fig. 5a). The transcriptional activities of TCTP 3′-UTR reporter showed that the overexpressed miR-425 or miR-371-5p partially downregulated TCTP expression with no statistic significant difference observed, and miR-125a-3p efficiently downregulated TCTP expression when compared with the miR-NC control conditions (Fig. 5b). To further confirm the regulation effect of miR-125a-3p on TCTP expression, miR-125a-3p inhibitor was transfected into the cells to inhibit miR-125a-3p. The transcriptional activities of TCTP 3′-UTR reporter showed that the miR-125a-3p inhibitor efficiently upregulated TCTP expression when compared with the miR-NC control, and facilitated TCTP expression in the cells treated by PM_2.5_ or NNK (Additional file 1: Figure S3D).

To confirm the results of miR125a-3p expression in vitro, we further evaluated the expression of miR-125a-3p in cell derived xenografts. Results revealed a significant decrease in the expression of miR-125a-3p in tumors formed from PM_2.5_ or NNK treated cells in comparison with that in tumors formed by the non-treated cells (Fig. 5c). We next examined the expression levels of human miR-125a-3p in 20 NSCLCs tumors and their paired non-tumors. The expression of human miR-125a-3p decreased in NSCLC tumors in comparison with matched non-tumors (p = 0.0271) (Fig. 5d). The mean levels of miR-125a-3p in NSCLCs were decreased approximate 4.0 folds of that in non-tumor (p = 0.036, Fig. 5e). Combined with the results of TCTP protein detection in human (Fig. 2), we could draw the conclusion that miR-125a-3p regulated TCTP expression negatively.

### Over-expressed miR-125a-3p significantly inhibited PM_2.5_- and NNK-induced EMT via down-regulated TCTP and vimentin

**Fig. 6:**
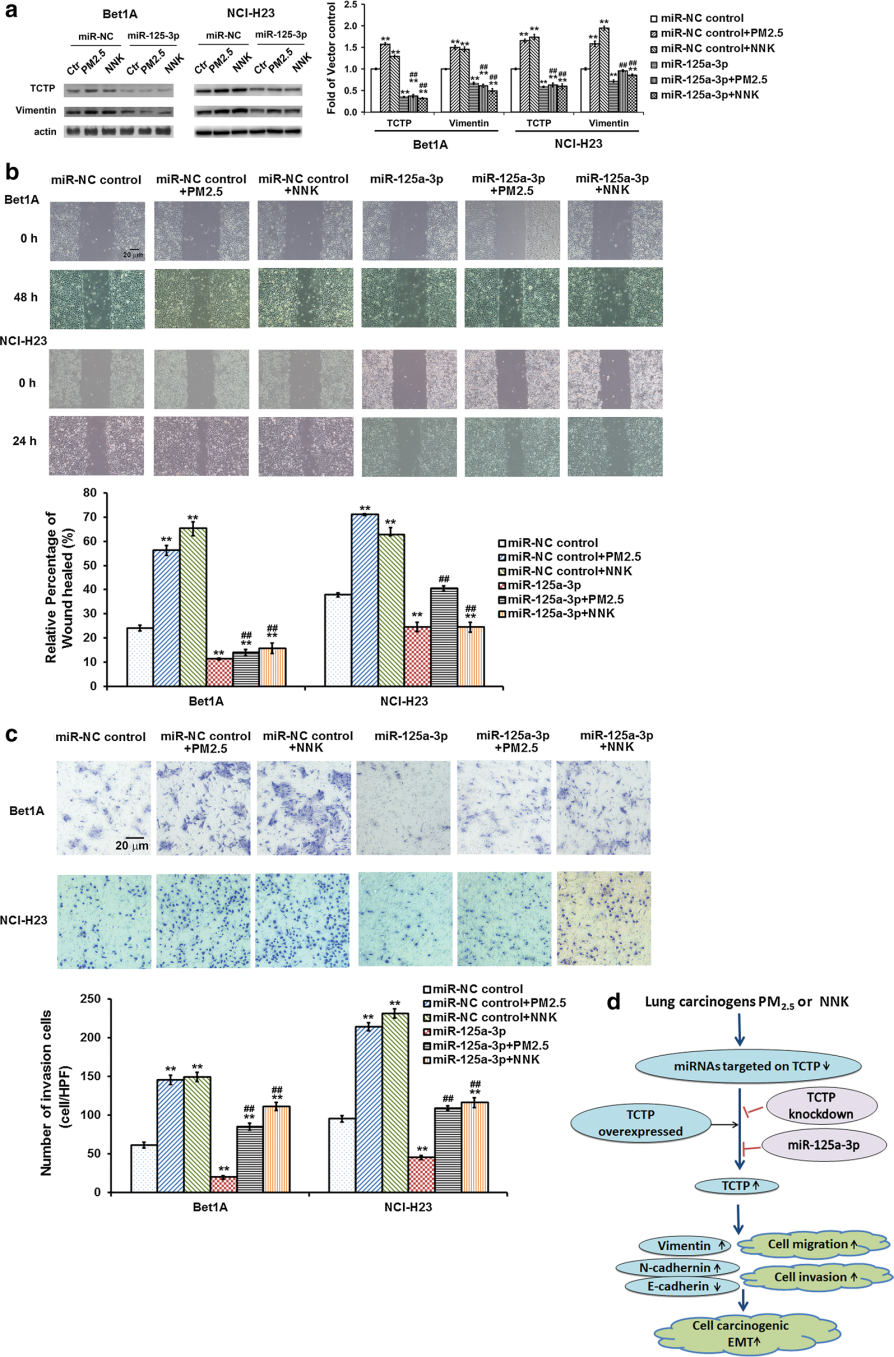
MiR-125a-3p inhibited PM_2.5_- and NNK-induced EMT via down-regulating TCTP. **a** Effect of miR-125a-3p on PM_2.5_- or NNK-induced TCTP and vimentin expression. After Bet1A or NCI-H23 cells were treated with PM_2.5_ or NNK for 28 days, the cells were transfected with miR-125a-3p mimic or miR-NC control respectively, cells were incubated for 24 h. The levels of TCTP and vimentin were determined. The equal loading was confirmed by measuring actin protein. The quantification of protein was carried out by densitometry analysis, and the result was presented by the relative intensity of the control condition based on actin normalization for total protein. The relative intensity of protein bands was summarized by column figure. The values indicate the mean ± SD of three independent experiments. **p < 0.01 vs each control condition; and ^##^*p *< 0.01 vs miR-NC + PM_2.5_ or miR-NC + NNK respectively. **b** Effect of miR-125a-3p on PM_2.5_- or NNK-induced cell migration. Bet1A cells and NCI-H23 cells were treated with PM_2.5_ or NNK for 28 days. Then the cells were transfected with miR-125a-3p mimic or miR-NC control respectively. Wound-healing assay were then performed. Images were taken using phase contrast microscope (Nikon) (scale bar, 20 μm). The values indicate the mean ± SD of three independent experiments, **p < 0.01 vs miR-NC control; ^##^*p *< 0.01 vs miR-NC + PM_2.5_ or miR-NC + NNK respectively. **c** Effect of miR-125a-3p on PM_2.5_- or NNK-induced cell invasion. Cells were treated by PM_2.5_ or NNK for 28 days. Then the cells transfected with miR-125a-3p mimic or miR-NC control respectively for invasion assay. Images were taken using phase contrast microscope (Nikon) (scale bar, 20 μm). The numbers of invading cells in four randomly selected high-power fields (HPF) were counted and the average number of cells in a HPF was calculated. The values indicate the mean ± SD of three independent experiments. **p < 0.01 vs miR-NC control; ^##^*p *< 0.01 vs miR-NC + PM_2.5_ or miR-NC + NNK respectively. **d** Schematic of the role of TCTP in promoting PM_2.5_- and NNK-induced EMT during lung carcinogenesis. Upon PM_2.5_ or NNK stimulation, the TCTP specific microRNA, namely miR-125a-3p, was down regulated, which in turn increased the expression of TCTP. Then TCTP promoted PM_2.5_- or NNK-induced EMT via up-regulating vimentin

Effect of over-expressed or knocked down of TCTP in PM_2.5_- and NNK-induced lung cells suggested that TCTP was involved in regulating vimentin expression during PM_2.5_- and NNK-mediated EMT (Fig. 3). And miR-125a-3p could function as the negative regulator of TCTP during this process (Figs. 4 and 5). We then assessed the over-expressed miR-125a-3p on PM_2.5_- and NNK-induced expression of TCTP and vimentin and lung cell EMT. When the mimic of miR-125a-3p was transfected into the cells, TCTP expression dropped and could not be elevated by PM_2.5_ and NNK treatment. Consistently, vimentin expression decreased when TCTP was downregulated by miR-125a-3p (Fig. 6a). Cells with over-expressed miR-125a-3p showed a lower migration and invasion potential even under PM_2.5_ or NNK treatment (Fig. 6b, c). The data indicated that TCTP was negatively regulated by miR-125a-3p.

## Discussion

Translationally controlled tumor protein was associated with a large number of cancer types including lung cancer [16, 17]. Tobacco product NNK and polluted air matter PM_2.5_ were two major contributors to lung carcinogenesis [2–4]. However, whether TCTP expression could be affected by PM_2.5_ or NNK in lung cells and if so, TCTP regulatory expression and its role in the process of lung carcinogenesis remained unclear. In this study, PM_2.5_- or NNK-treated lung epithelial and NSCLC cells, cell-derived xenografts together with human lung cancer samples were analyzed. We found that TCTP expression was up-regulated during PM_2.5_ or NNK exposure and required for lung cell EMT.

Long-term cell cultures have been used to study lung tumorigenesis [30, 31]. In this study, we treated the lung epithelial cell Bet1A and NSCLC cell NCI-H23 with PM_2.5_ or NNK for a period of 28 days to induce lung cell carcinogenesis. In this case, both NNK and PM_2.5_ treatment could increase TCTP accompanied with cell acquiring EMT characteristics. After long-time exposure to PM_2.5_ or NNK, transient TCTP knockdown could effectively eliminate the EMT induced by these two carcinogens (Fig. 3 and Additional file 1: Figure S1). The role of TCTP was further demonstrated by its high expression level in NCI-H23 cell derived xenografts (CDX) and human NSCLCs lung tumors (Figs. 1 and 2). When we tried to construct TCTP-shRNA stable expression cells to complete CDX experiment, we failed to acquire enough healthy NCI-H23 cells that contained stable expression of TCTP-shRNA. Because cell viability decreased while cell death increased during the process of TCTP-shRNA-cell line selection, it supported the notion that homozygous mutants of TCTP were embryonic lethal [32]. Thus, TCTP exhibited the function of housekeeping genes to some extent. Our work was not consistent with Wang’s report that tumors formed after in vivo TCTP silencing [33]. The discrepancy might due to different cell lines that applied for the experiment (NCI-H23 vs A549).

No matter in PM_2.5_/NNK treated Bet1A and NCI-H23 or in the cell derived xenografts and human NSCLCs lung tumors, high expression of vimentin was accompanied with high level of TCTP (Figs. 1, 2). When TCTP was overexpressed, the expression of vimentin was increased. When TCTP was knocked down, vimentin level decreased even in the presence of PM_2.5_/NNK (Fig. 3). The results showed that TCTP controlled the expression of vimentin, which demonstrated again that TCTP controlled carcinogens-induced EMT. And our work also supported the notion that TCTP expression was required for TGF-β1 induced EMT in A549 lung cancer cells [17]. Our recently study found that PM_2.5_, as well as NNK [27], increased the expression of β-catenin, pGSK3β and TCF4 and activated Wnt/β-catenin signal pathway. In lung cancer cells, interaction of TCTP with β-catenin stabilized β-catenin and facilitated translocation of β-catenin to the nucleus in order to activate EMT regulators [17]. Based on these studies, we could speculate that TCTP regulated vimentin expression and EMT through β-catenin and Wnt/β-catenin signal pathway in lung carcinogen induced lung cell EMT. Furthermore, our correlation analysis showed that high levels of TCTP and vimentin were significantly associated with tumor size and age respectively but not with other clinicopathological features. Thus, high levels of TCTP and vimentin might act as tumor enhancer for NSCLCs in both smokers and non-smokers (Additional file 1: Table S1). The results were consistent with the report that high TCTP levels were typically associated with advanced tumors and poor patient outcomes of brain tumor, breast cancer, colorectal cancer, hepatocellular carcinoma, neuroblastomas, and ovarian cancer [28].

Although TCTP was required for carcinogens-induced EMT, how PM_2.5_ and NNK up-regulated TCTP expression remained unclear. Transcriptional regulation of TCTP gene via transcription factor such as CREB, P53, and HIF-1α has been reported [28], however, DNA methylation, the most studied epigenetic regulatory mechanism on transcriptional level, was not yet examined for TCTP. In our experiments, lower level of DNA methylation in TCTP promoter region and the discrepancy between NCI-H23 and Bet1A cells indicated that methylation did not play a key role in TCTP expression in PM_2.5_ and NNK induced EMT (Additional file 1: Figure S2). Except for DNA methylation, post-transcriptional regulation by miRNAs is also involved in fundamental biological processes. MicroRNAs could negatively regulate gene expression by binding to 3′-UTR of target mRNAs; causing translational repression or degradation of target mRNAs [34]. For example, miR-27b was proved to negatively regulate TCTP in oral cancer [35]. But there was no report on the regulation of TCTP expression by miRNA in lung cancer. Our luciferase assay of lung cells transfected with 3′-UTR of TCTP plasmids have demonstrated that TCTP expression could be effectively regulated by microRNAs. Real-time PCR results showed that three miRNAs, miR-125a-3p, miR-425 and miR-371-5p, which potentially targeted on TCTP 3′-UTR region, were significantly decreased by PM_2.5_ or NNK treatment. Among these three miRNAs, MiR-125a-3p expression levels has been reported to be lower in NSCLC tissues when compared with normal lung tissues and were associated with poor prognoses in NSCLC [36]. MiR-125a-3p inhibited the proliferation, migration, and invasion of NSCLC cells [37, 38]. MiR-371-5p played an important role of “oncosuppressor” in colorectal cancer progression in the regulation of EMT, stemness and metastasis [39]. As for miR-425, it was also a potential tumor suppressor in cancer to inhibit cell proliferation and metastasis and induced cell apoptosis [40]. Our results were highly consistence with these reports. To further confirm the effect of the three miRNAs, we over-expressed these three microRNAs in PM_2.5_- and NNK-induced lung cells to check the TCTP expression and EMT. Intriguingly, only miR-125a-3p overexpression significantly counteracted the PM_2.5_- and NNK-induced TCTP expression and lung cancer cell EMT. It was well known that miR-125a-3p might target some genes and reduce their levels. MiR-125a-3p acted as a tumor suppressive miRNA in various human malignancies such as malignant glioma, prostate, gastric, pancreatic and lung cancer [36, 41–44]. It was reported that miR-125a-3p decreased the expression of RhoA to represses cell migration of lung cancer cells [45]. miR-125a-3p down-regulated Fyn and Fyn-downstream genes, and inhibited prostate cancer cells proliferation and migration [42]. Nrg1 was directly regulated by miR-125a-3p, which in turn led to the inhibition of glioma cell proliferation and invasion [46]. In this report, we provided the evidence for the first time that miR-125a-3p impacted the malignant lung cell EMT by binding with the 3′-UTR of TCTP, which in turn decreased the protein level of TCTP followed by down-regulating vimentin expression. The data indicated that miR-125a-3p down-regulation significantly contributed to TCTP overexpression in PM_2.5_- and NNK-induced lung cancer cell EMT. Knockdown of TCTP could imitate the role of miR-125a-3p overexpression in suppressing lung carcinogens induced malignant EMT. Considering that this miRNA might target different genes, we could not exclude the possibility that miR-125a-3p might also exhibit its EMT suppressive effect via down-regulating the expression of genes other than TCTP. Given the evidence that TCTP knockdown alone could block the effect of PM_2.5_- and NNK stimulation, it was reasonable that miR-125a-3p mainly inhibited EMT through TCTP.

## Conclusions

In summary, it was the first trial to experimentally investigate TCTP regulatory expression and its function in the process of lung carcinogenesis induced by lung carcinogens PM_2.5_ or NNK. Our findings suggested a very important role of TCTP in promoting PM_2.5_- and NNK-induced EMT during lung carcinogenesis (Fig. 6d). TCTP controlled EMT via regulating vimentin expression. TCTP level could be regulated the microRNA miR-125a-3p. Our work results exhibited that TCTP and miR-125a-3p were important prognostic biomarkers for NSCLC and potential targets for clinical therapy.

## Supplementary information

**Additional file 1: Figure S1.** TCTP controlled vimentin expression and mediated PM_2.5_- and NNK-induced metastasis of lung cells. **Figure S2**. MassArray for TCTP methylation detection. **Figure S3.** (A-C) Cloning of TCTP 3′-UTR. And (D) TCTP transcriptional activity was upregulated by miR-125a-3p inhibitor. **Table S1.** Baseline demographic characteristics of 109 human NSCLC patients underwent TCTP and vimentin analysis. **Table S2.** Human TCTP gene methylation level in NCI-H23 and Bet1A cells treated by PM2.5 and NNK.

## Data Availability

All data generated or analyzed during this study are included in this manuscript and the online additional files.

## Abbreviations

CDX: Cell derived xenografts
CSC: Cancer stem cell
DMEM: Dulbecco’s modified Eagle medium
EMT: Epithelial–mesenchymal transition
FBS: Fetal bovine serum
IHC: Immunohistochemistry
LCNS: Lung cancer in never smokers
NNK: 4-Methylnitrosamino-l-3-pyridyl-butanone
NSCLC: Non-small cell lung cancer
PM_2.5_: Particulate matter 2.5
OS: Overall survival
PBS: Phosphate-buffered saline
RFS: Recurrence-free survival
S.C.: Subcutaneous
TCTP: Translationally controlled tumor protein
UTR: Untranslated region
