# Supplementary material for: The permissive role of TCTP in PM2.5/NNK-induced epithelial–mesenchymal transition in lung cells
Source: J Transl Med. 2020 Feb 11;18:66. doi: 10.1186/s12967-020-02256-5 (PMC7011287; doi:10.1186/s12967-020-02256-5)
Supplement: Supplementary file 1 — Additional file 1: Figure S1. TCTP controlled vimentin expression and mediated PM2.5- and NNK-induced metastasis of lung cells. Figure S2. MassArray for TCTP methylation detection. Figure S3. (A-C) Cloning of TCTP 3′-UTR. And (D) TCTP transcriptional activity was upregulated by miR-125a-3p inhibitor. Table S1. Baseline demographic characteristics of 109 human NSCLC patients underwent TCTP and vimentin analysis. Table S2. Human TCTP gene methylation level in NCI-H23 and Bet1A cells treated by PM2.5 and NNK. [file 12967_2020_2256_MOESM1_ESM.pdf]

The permissive role of TCTP in $PM_{2.5}$/NNK-induced epithelial-mesenchymal transition in lung cells

Running title: TCTP controls carcinogenic EMT in lung cells

Li-Zhong Liu[1*], Menghuan Wang[1], Qihang Xin[1], Bowen Wang[1], George G Chen[2, 4*], Ming-Yue Li[2, 3,4*]

[1]Guangdong Provincial Key Laboratory of Regional Immunity and Diseases, Department of Physiology, School of Medicine, Shenzhen University Health Science Center, Shenzhen University, Shenzhen, China.

[2]Department of Surgery, The Chinese University of Hong Kong, Prince of Wales Hospital, Shatin N.T., Hong Kong.

[3]Guangzhou Regenerative Medicine and Health Guangdong Laboratory, Guangzhou, China

[4]Shenzhen Research Institute, The Chinese University of Hong Kong, Shenzhen, Guangdong, China.

[*]**Corresponding author**: Ming-Yue Li or George G Chen, Department of Surgery, Prince of Wales Hospital, Shatin N.T., Hong Kong. Tel: 852 37636161; Fax: 852 26450605; Email: brightmoonli@yahoo.com or gchen@cuhk.edu.hk; or Li-Zhong Liu, Faculty of Medicine, Shenzhen University Health Science Center, Shenzhen University, Shenzhen, China. Tel: 86-755-86671936; Email: liulz@szu.edu.cn.

Additional file 1 information:

1. Figures S1-S3
2. Tables S1-S2

Figure S1

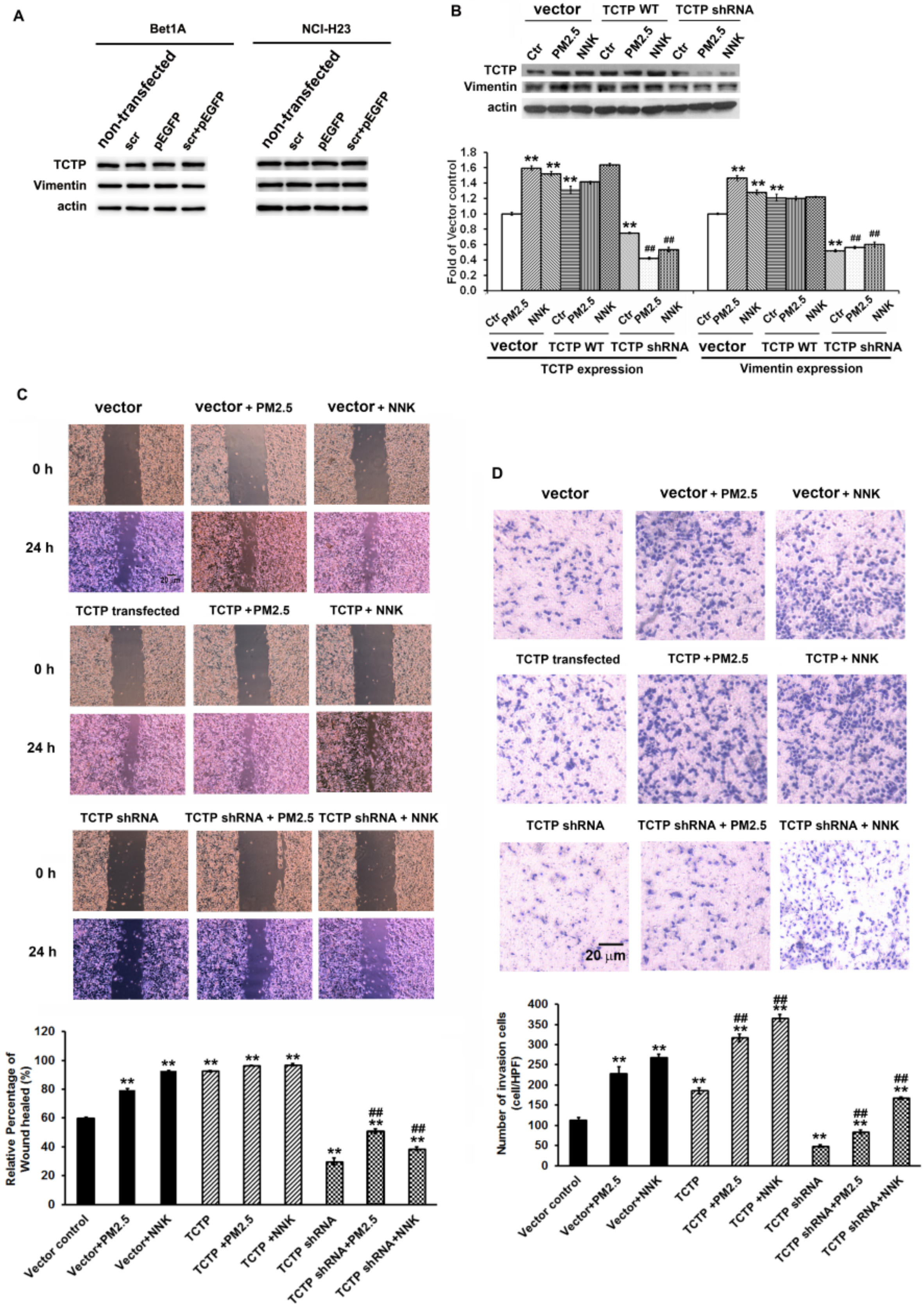

A
Bet1A
NCI-H23
non-transfected
scr
pEGFP
scr+pEGFP
TCTP
Vimentin
actin
B
vector
TCTP WT
TCTP shRNA
Ctr
PM2.5
NNK
Fold of Vector control
TCTP expression
Vimentin expression
C
vector
vector + PM2.5
vector + NNK
0 h
24 h
TCTP transfected
TCTP +PM2.5
TCTP + NNK
TCTP shRNA
TCTP shRNA + PM2.5
TCTP shRNA + NNK
Relative Percentage of Wound healed (%)
Vector control
Vector+PM2.5
Vector+NNK
TCTP
TCTP +PM2.5
TCTP +NNK
TCTP shRNA
TCTP shRNA+PM2.5
TCTP shRNA+NNK
D
20 μm
Number of invasion cells (cell/HPF)

Figure S1. TCTP controlled Vimentin expression and mediated $PM_{2.5}$- and NNK-induced metastasis of lung cells. (A) TCTP and Vimentin expression in cells transfected with different vectors. Bet1A or NCI-H23 cells were transfected with scramble shRNA pSicoR vector (Scr), empty pEGFP vector, or scramble shRNA pSicoR vector + empty pEGFP vector (Scr+pEGFP) respectively for 24 h. Non-transfected cells were set up as control. The levels of TCTP and vimentin were determined as indicated. The equal loading was confirmed by measuring actin protein. (B) TCTP controlled vimentin expression. NCI-H23 cells were first treated with $PM_{2.5}$ or NNK for 28 days, then the cells were co-transfected with scramble shRNA pSicoR vector + empty pEGFP vector or cells were transfected with pSicoR vector containing TCTP shRNA or pEGFP vector containing TCTP gene respectively. The levels of TCTP and vimentin were determined as indicated. The equal loading was confirmed by measuring actin protein. The quantification of protein was carried out by densitometry analysis, and the result was presented by the relative intensity of the control condition based on actin normalization for total protein. The relative intensity of protein bands was summarized by column figure. The values indicate the mean ± SD of three independent experiments (**$p<0.01$ vs vector control; $^{\#\#}p<0.01$ vs vector+$PM_{2.5}$ or vector+NNK respectively). **(C) TCTP was required for $PM_{2.5}$- or NNK-induced cell migration.** NCI-H23 cells were treated with $PM_{2.5}$ or NNK for 28 days. Then the cells were transfected with empty vector or vector contained TCTP gene or TCTP shRNA respectively. Cell migration was detected by wound-healing assay. Images were taken using phase contrast microscope (Nikon) (scale bar, 20 μm). The relative percentage of wound healed was expressed as the values indicated the mean ± SD of three independent experiments. **$p<0.01$ vs vector control; $^{\#\#}p<0.01$ vs vector+$PM_{2.5}$ or vector+NNK respectively. **(D) TCTP was required for $PM_{2.5}$- or NNK-**

induced cell invasion. NCI-H23 cells were treated by $PM_{2.5}$ or NNK for 28 days. Then the cells transfected with vectors as indicated. Cell invasion was detected by trans-well experiment. Images were taken using phase contrast microscope (Nikon) (scale bar, 20 μm). The numbers of invading cells in four randomly selected high-power fields (HPF) were counted and the average number of cells in a HPF was calculated. The values indicate the mean ± SD of three independent experiments. $^{**}p<0.01$ vs vector control; $^{\#\#}p<0.01$ vs vector+$PM_{2.5}$ or vector+NNK respectively.

Figure S2A

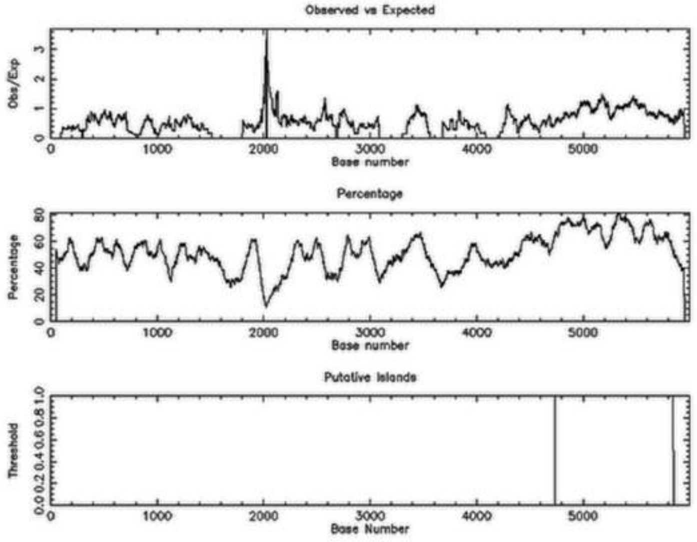


```
CPGPLOT islands of unusual CG composition
EMBOSS_001 from 1 to 6000

     Observed/Expected ratio > 0.60
     Percent C + Percent G > 50.00
     Length > 200

 Length 1110 (4738..5847)
```

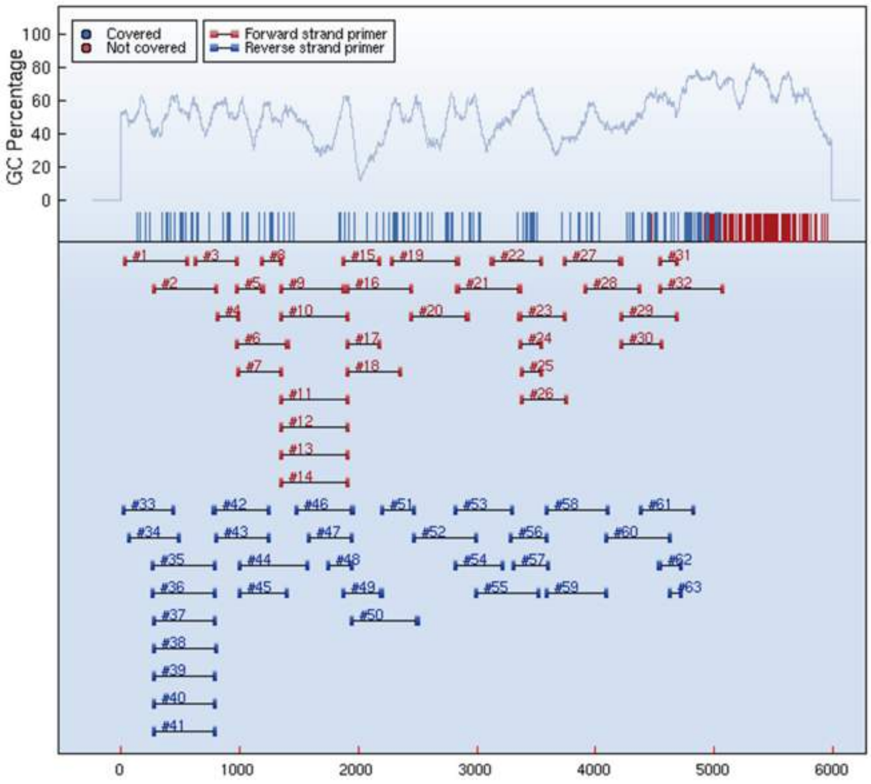

Figure S2B

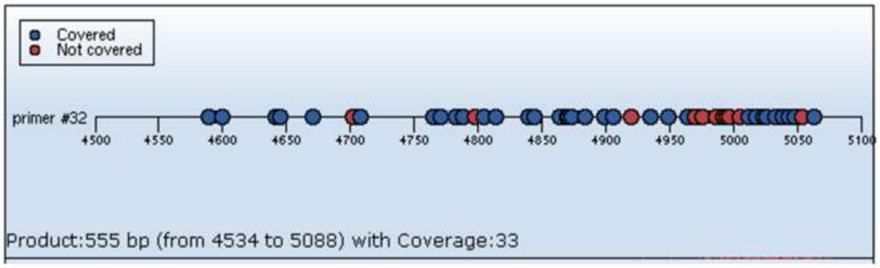

aaaactccagctggggcagccctggggcagatgctgaaaagttgtcagaggccctCGggcagtccCGagatctaccccaggccagagggcctgaccctccct
aaatgCGaCGttctcctaccttggttgatactcaCGttcccagaaaagggtggaacctaggctggaCGaggCGcagggccaaagtttaattcctctaagctc
cacccagctcccagcacctctccaggCGgcccCGtggggtagggCGgagcCGggtccaaCGtactcCGcttccccCGctccacccacccagggctaggg
agCGcccCGagagttggcctcctcccaCGctgCGCGCGcacctcccCGcccccacccctaccCGctggCGtgcccagtggaaCGgagccttgtgtctc
CGcctcaagtcccCGgatgctcacctcccCGactCGccccCGctgtggcccCGccccCGCGCGgctCttCGtgccaCGtcacCGcctgCGtCGcttc
CGgaggCGcagCGggCGatgaCGtagagggaCGtgccctctatatgaggttggggag

Primer	Start	Size	Tm	GC%	C's
5′ **end**	4534	25	59.94	32	8
3′ **end**	5088	25	61.10	40	4
PRODUCT Size:555, No of CpG's : 44, Coverage : 33					

5′ : aggaagagagAAAATTTTAGTTGGGGTAGTTTTGG
3′ : cagtaatacgactcactatagggagaaggctCTCCCCAACCTCATATAAAAAACAC

Figure S2C

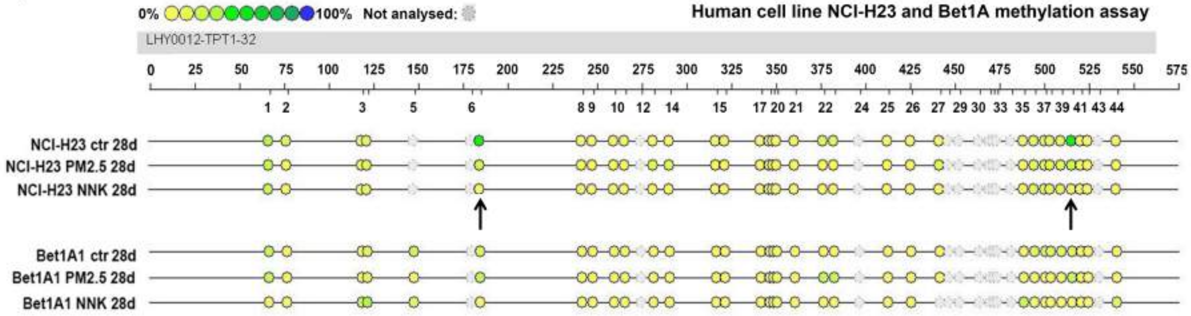

Figure S2. MassArray for TCTP methylation detection. (A) Prediction of potential CpG islands by linking to http://www.ebi.ac.uk/Tools/seqstats/emboss_cpgplot/. The CpG islands from 5000bp upstream of start codon to 1000bp downstream of start codon of TCTP genes were predicted by the software www.ebi.ac.uk/Tools/seqstats/emboss_cpgplot/. One potential CpG island was found which located from 4738-5847 bp (Length 1110 bp) of the 6000 bp sequence analyzed. **(B) Primers design using sequenom®EpiDesigner program.** Plan #32 was recommended and selected for the methylation assay. One CpG island found in TCTP promotor

region was analyzed by Plan #32 (coverage of 555bp from 4534 to 5088) of the promotor. **(C) Change of TCTP promotor methylation after $PM_{2.5}$ or NNK treatment.** NCI-H23 and Bet1A cells were treated with $PM_{2.5}$ and NNK respectively for 28 d. Then the methylation of CpG islands was examined. For cancer cell line NCI-H23, among the 44 CpG points detected, only two points of CpG islands (CpG 7 and CpG 40) exhibited a 42% methylation in basal state. The methylation reduced 50% after PM2.5 treatment for 28d while the methylation disappeared after NNK treatment (also refer to Table S2). The two sites with the greatest change in methylation level were labeled with arrows. For normal lung epithelial cell Bet1A, there was only 17% methylation on the same CpG islands (CpG 7 and CpG 40) in basal state. The methylation reached 30% after cells were treated with PM2.5 whereas NNK exposure eliminated the methylation (also refer to Table S2).

Figure S3

A

Xbal in PGL3-promotor-vector and
Six selected restriction enzymes

name	*sequence*
XbaI	5'...TCTAGA...3'<br>3'...AGATCT...5'
AgeI	5'...ACCGGT...3'<br>3'...TGGCCA...5'
ApaI	5'...GGGCCC...3'<br>3'...CCCGGG...5'
EcoRI	5'...GAATTC...3'<br>3'...CTTAAG...5'
NdeI	5'...CATATG...3'<br>3'...GTATAC...5'
PstI	5'...CTGCAG...3'<br>3'...GACGTC...5'
SpeI	5'...ACTAGT...3'<br>3'...TGATCA...5'

Sequences for cloing MCS into the vector:

Forward (5'-3'): CTAGAACACTAGTTGCTGCAGACCATATGTCGAATTCAGGGGCCCTGACCGGTACT

Reverse (5'-3'): CTAGAGTACCGGTCAGGGCCCCTGAATTCGACATATGGTCTGCAGCAACTAGTGTT

Orientation of MCS in the vector confirmed by sequecing: ***SpeI-PstI-NdeI-EcoRI-ApaI-AgeI***

B

TCTP 3'-UTR

```
  1 CAAAUGUGGCAAUUAUUUUGGAUCUAUCACCUGUCAUCAUAACUGGCUUCUGCUUGUCAUCCACACAACACCAGG 75
 76 ACUUAAGACAAAUGGGACUGAUGUCAUCUUGAGCUCUUCAUUUAUUUUGACUGUGAUUAUUUGGAGUGGAGGCA 150
151 UUGUUUUUAAGAAAAACAUGUCAUGUAGGUUGUCUAAAAAUAAAAUGCAUUUAAACUCAUUUGAGAG         217
```

C

Corresponding sequence of cDNA to 3'-UTR of TCTP

```
601 ggaaaaatgt taacaaatgt ggcaattatt ttggatctat cacctgtcat cataactggc
661 ttctgcttgt catccacaca acaccaggac ttaagacaaa tgggactgat gtcatcttga
721 gctcttcatt tattttgact gtgatttatt tggagtggag gcattgtttt taagaaaaac
781 atgtcatgta ggttgtctaa aaataaaatg catttaaact catttgagag
```

Primers: (PCR product size 235bp)

TCTP-UTR_EcoRI-F: GGT G'AATTC CAA ATG TGG CAA TTA TTT TGG

TCTP-UTR_AgeI-R: CCT A'CCGGT CTC TCA AAT GAG TTT AAA TGC

D

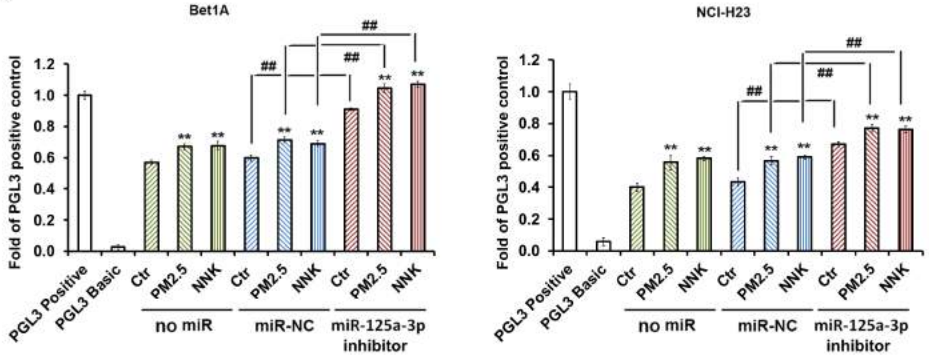

Figure S3. (A-C) Cloning of TCTP 3'-UTR. The Luc-TCTP-WT with full-length 3'-untranslated region (UTR) of TCTP (217 nt) were cloned into pGL3-promoter-vector. **(A) Reconstruction of pGL3-promoter-vector.** pGL3-promoter-vecotr contains XbaI site at the end of luciferase gene. Six restriction enzymes that do not cut the pGL3-promoter-vector, *AgeI, ApaI,*

EcoRI, NdeI, PstI and SpeI, were selected. A newly designed MCS (multiple cloning site) within XbaI site was inserted, which allows cloning of control element (e.g. 3'-UTR) into pGL3-promoter after Luc gene. The orientation of MCS was *SpeI-PstI-NdeI-EcoRI-ApaI-AgeI* as confirmed by sequencing after cloning. **(B) The sequence of TCTP 3'-URT. (C) Cloning of TCTP 3'-UTR into the pGL3-promoter-vector.** The cDNA fragment of TCTP (https://www.ncbi.nlm.nih.gov/nuccore/555943819) was amplified by reverse-transcription PCR using the following primer: TCTP-UTR_EcoRI-Forward GGTG'AATTCCAAATGTGGCAATTATTTTGG and TCTP-UTR_AgeI-Reverse CCTA'CCGGTCTCTCAAATGAGTTTAAATGC. The PCR products were 235bp. **(D) TCTP transcriptional activity was upregulated by miR-125a-3p inhibitor.** Bet1A cells and NCI-H23 cells were treated with $PM_{2.5}$ or NNK for 28 days and were con-transfected with miR-125a-3p inhibitor and miR-NC (#339121 miRCURY LNA miRNA Inhibitor, Qiagen MD) respectively with luciferase reporter constructs containing the pGL3-TCTP 3'-UTR. The pGL3 basic vector and the pGL3 control were used as negative and positive controls respectively. Reporter assays were performed using the Dual-luciferase assay system, normalized for transfection efficiency by co-transfected Renilla luciferase. Data was expressed as mean ± SD of three independent experiments performed in triplicate. $^{**}p<0.01$ vs control, and $^{\#\#}p<0.01$ when compared between indicated groups.

Table S1. Baseline demographic characteristics of 109 human NSCLC patients underwent TCTP and Vimentin analysis.

Characteristics	Tumor TCTP expression			Tumor Vimentin expression		
	High level	Normal level	p	High level	Normal level	p
**Overall**	68	41	< 0.01	61	48	< 0.001
**Age**						
**≤65**	31	14		30	14	
**>65**	37	27	0.3157	31	34	0.0345
**Gender**						
**Male**	50	25		42	35	
**Female**	18	16	0.0844	19	13	0.8985
**Smoking status**						
**smoker**	25	9		17	17	
**ex-smoker**	20	16		18	18	
**non-smoker**	23	16	0.2582	26	13	0.2438
**Histology**						
**squamous cell carcinoma**	19	8		15	12	
**adenocarcinoma**	40	25		37	28	
**large cell carcinoma**	5	1		2	4	
**poorly differentiated carcinoma**	4	7	0.1682	7	4	0.6741
**Tumor size**						
**<50mm**	47	20		36	33	
**50mm≥**	21	21	0.0431	25	15	0.3230
**Pathology stage**						
**I-II**	52	30		49	36	
**III-IV**	16	11	0.8193	16	11	0.4934

Table S2. Human TCTP gene methylation level in NCI-H23 and Bet1A cells treated by PM2.5 and NNK.

Sample ID	CPG Position	NCI-H23			Bet1A1		
		ctr 28day	NNK 28 day	PM2.5 28day	ctr 28day	NNK 28 day	PM2.5 28day
LHY0012-TPT1-32_CpG_1	66	0.33	0.24	0.23	0.26	0.09	0.29
LHY0012-TPT1-32_CpG_2	76	0.16	0	0	0	0	0
LHY0012-TPT1-32_CpG_3	118	0	0	0	0	0.28	0.03
LHY0012-TPT1-32_CpG_4	121	0.16	0.22	0.15	0.15	0.33	0.19
LHY0012-TPT1-32_CpG_5	147	NA	NA	NA	0.29	0.19	0.1
LHY0012-TPT1-32_CpG_6	179	NA	NA	NA	NA	NA	NA
LHY0012-TPT1-32_CpG_7	184	0.42	0	0.21	0.17	0	0.3
LHY0012-TPT1-32_CpG_8.9	241:247	0	0.09	0.02	0.04	0.03	0
LHY0012-TPT1-32_CpG_10	259	0	0	0	0	0	0
LHY0012-TPT1-32_CpG_11	265	0	0.05	0	0	0	0
LHY0012-TPT1-32_CpG_12	274	NA	NA	NA	NA	NA	NA
LHY0012-TPT1-32_CpG_13.14	281:290	0.05	0.06	0.19	0.02	0	0.02
LHY0012-TPT1-32_CpG_15.16	316:321	0	0	0	0	0	0
LHY0012-TPT1-32_CpG_17.18.19.20	341:346:348:350	0.06	0.04	0.05	0.07	0.02	0.02
LHY0012-TPT1-32_CpG_21	360	0	0.08	0	0	0.05	0
LHY0012-TPT1-32_CpG_22.23	376:382	0.16	0.1	0.03	0.08	0.06	0.23
LHY0012-TPT1-32_CpG_24	396	NA	NA	NA	NA	NA	NA
LHY0012-TPT1-32_CpG_25	412	0.09	0	0	0.03	0	0
LHY0012-TPT1-32_CpG_26	425	0	0	0	0	0	0
LHY0012-TPT1-32_CpG_27	441	0	0	0.19	0.01	NA	0.09
LHY0012-TPT1-32_CpG_28.29.30.31.32.33.34	:469:471:473:481	NA	NA	NA	NA	NA	NA
LHY0012-TPT1-32_CpG_35	488	0.05	0	0	0	0.28	0
LHY0012-TPT1-32_CpG_36.37.38.39	494:500:503:509	0.12	0.11	0.13	0.11	0.07	0.01
LHY0012-TPT1-32_CpG_40	515	0.42	0	0.21	0.17	0	0.3
LHY0012-TPT1-32_CpG_41.42	520:524	0.02	0.02	0.03	0	0	0.01
LHY0012-TPT1-32_CpG_43	530	NA	NA	NA	NA	NA	NA
LHY0012-TPT1-32_CpG_44	540	0	0	**0**	0	0.14	0

Note: Same color of CpG site means that the length of PCR products are indetical and the methylation unit presented are the mean of the two sites' methylation level.
